# Predictors of response and drug survival in ankylosing spondylitis patients treated with infliximab

**DOI:** 10.1186/s12891-015-0620-4

**Published:** 2015-07-24

**Authors:** Mariagrazia Lorenzin, Augusta Ortolan, Paola Frallonardo, Francesca Oliviero, Leonardo Punzi, Roberta Ramonda

**Affiliations:** Rheumatology Unit, Department of Medicine – DIMED, University of Padova, Via Giustiniani 2, 35128 Padova, Italy

**Keywords:** Spondyloarthritis, Ankylosing spondylitis, anti-TNFα agents, Biologic drugs, Infliximab

## Abstract

**Background:**

The advent of anti-tumor necrosis factor-α (TNFα) drugs has changed the course of ankylosing spondylitis (AS). While data are available concerning the long term effectiveness of single anti-TNF agents, little has been published about predictors of treatment response in AS. The aim of this retrospective study was to evaluate the survival, effectiveness, and safety of infliximab over a 5-year period and to identify predictors of disease outcome.

**Methods:**

Seventy AS patients attending the Rheumatology Clinic of the University of Padua who were treated with intravenous infliximab at 0, 2, 4 weeks and then every 6, 8, or up to 16 weeks were studied retrospectively. Demographic information, laboratory inflammatory and disease indices (BASDAI, BASFI, BASMI) were collected (at baseline, 3, 6, 12 months and once a year thereafter). Clinical improvement, drug tolerability, adverse events/side effects and causes leading to discontinuation were recorded.

**Results:**

Infliximab caused a rapid, persistent improvement at all the assessment times in the BASDAI 50 (71.4 %) and ASDAS scores (97.1 % in ASAS20, 80 % in ASAS40, 80 % in ASAS5/6), and already within 6 months of beginning treatment in 50 % percent of the patients. The other 50 % withdrew because of: adverse events (12 = 34.3 %), side effects (5 = 14.3 %), drug inefficacy (12 = 34.3 %), spontaneously (4 = 11.4 %). Those who did not respond were prevalently females (34.3 % vs 17.1 %).

**Conclusion:**

Factors such as female sex, use of steroids, persistently high inflammatory levels, BASFI and BASDAI indices were found to be negative predictors of treatment response. Infliximab was found to be safe, effective and well-tolerated; it elicited satisfactory long term response and drug survival rates.

## Background

Ankylosing spondylitis (AS), a prototype of the spondyloarthritis (SpA) family, is a chronic, progressive, axial inflammatory disease mainly involving the spine and the sacroiliac joints [1], as well as other sites of the axial skeleton such as anterior chest wall joints [2]. Since the disease can lead to severe, chronic pain and discomfort [1, 3], treatment should be initiated as early as possible to prevent skeletal deformity and physical disability [4]. In the past (until’90s), few effective therapies were available and were limited for the most part to regular physical activity and non-steroidal anti-inflammatory drugs (NSAIDs) [1, 5, 6]. Disease-modifying antirheumatic drugs (DMARDs) and corticosteroids, which are quite effective in other chronic inflammatory diseases such as rheumatoid arthritis (RA), appear to have only a limited or no effect on the course of AS [1, 6–9].

The advent of anti-Tumor Necrosis Factor-α (TNFα) agents (adalimumab, etanercept, infliximab and golimumab) has constituted a breakthrough in the management of AS patients, especially in the case of persistently active forms [1, 10–17]. Anti-TNFα agents have been found to produce clinical, functional, biological, as well as quality of life improvements. Trials with infliximab and other anti-TNFα agents have, in fact, demonstrated a remarkable improvement in AS patients, and their efficacy and safety have been confirmed by a number of randomised, open-label, controlled studies [17–22]. These results need, however, to be confirmed by further studies focusing on some treatment-related aspects and patients’ real life experience. While single center studies have many limitations with respect to multicenter studies or registries, they have the advantage of studying a more homogeneous population and of thus preventing at least one confounding factor. The aim of this retrospective single center study was to evaluate the effectiveness, safety and the long-term drug survival of infliximab in AS patients over a 5-year treatment period and to identify predictors of disease outcome.

## Methods

### Study population

Seventy AS outpatients diagnosed between 2003 and 2010 in accordance with the modified New York criteria [23] and attending the Rheumatology Clinic of the University of Padova Medical Center were enrolled in this retrospective study. This study was approved by the ethics committee of Padova University Hospital. All of the patients provide written informed consent for participation in this study. Inclusion criteria were: age >18 years and an inadequate response (no response or lack of tolerance) to previous NSAIDs. Exclusion criteria were: signs or symptoms of latent or active tuberculosis, chronic or clinically severe infection, malignancy or congestive heart failure.

All the recruited patients were treated with infliximab, which was administered intravenously (3–5 mg/kg/body weight) at 0, 2, 6 weeks and every 4 weeks thereafter for a 5-year period. Whenever drug scheduling produced an optimal treatment response, the time interval between drug infusion was prolonged to 6, 8 or to as many as 16 weeks. In addition to pharmacological therapy, all patients followed a home-based exercise program recommended by the specialized physiotherapist who collaborates with our unit.

### Clinimetric, laboratory tests, assessment questionnaires, and outcome measures

All the patients underwent regular clinical and clinimetric evaluations prescribed by a specialized rheumatologist. Demographic data and assessment scores at baseline (0 M), at 3 months (3 M), at 6 months (6 M), and once a year thereafter for a 5-year period were systematically collected.

The instruments utilized included the Bath Ankylosing Spondylitis Disease Activity Index (BASDAI) which is the most commonly used instrument to measure and evaluate the inflammatory activity of AS (values expressed from 0 to 100). Considered the gold standard to assess physical function in AS patients and commonly used to screen candidates for anti-TNF medication the Bath Ankylosing Spondylitis Functional Index (BASFI), is a self-reported, validated, outcome measure. Active disease was identified by the BASDAI and/or BASFI score > 4 [24, 25]. Spinal mobility was measured using the Bath Ankylosing Spondilitis Metrology Index (BASMI). Pain and global assessment were measured using the Visual Analogue Scale and patient global assessment (VAS and VASg) whose scores range from 0 to 10. Laboratory measures of inflammation, such as C-reactive protein (CRP) and erythrocyte sedimentation rate (ESR), were regularly assessed in all the patients (normal values for our laboratory: <0.6 mg/dL for CRP and <20 mm/hour for ESR). Clinical improvement in the patients who continued to receive infliximab throughout the study period was also assessed using the Ankylosing Spondylitis Assessment Study Group (ASAS) criteria 20 and 40 % and the ASAS 5/6 response criteria [26]. The ASAS20 defines improvement as at least 20 % (or an absolute improvement of at least 10 units on a scale of 0–100) in three of the following domains: patient’s global assessment of disease activity, pain, function (BASFI score) and morning stiffness (considered the average of the last two questions on the six-question BASDAI regarding morning stiffness). The ASAS40 defines improvement as at least 40 % or an absolute improvement of at least 20 units on a 0–100 mm scale in at least three out of the four domains, without worsening in the remaining one. The “ASAS five out of six criteria” defines improvement as a 20 % amelioration in any of five of the following six domains: the four domains used for ASAS 20 % and ASAS 40 %, acute phase reactants and spinal mobility, as assessed by the BASMI score.

### Statistical analysis

The Mann–Whitney *U* test was used to compare the clinical (BASDAI, BASFI, BASMI, VAS, VASg) and inflammatory indices (CRP, ESR) of the patients who continued to take infliximab throughout the study period and those who did not at time 0 (0 M) and 60 months later (60 M). The Friedman Repeated Measures test followed by the Dunn's Multiple Comparison Test were used to evaluate the pattern in these parameters over time (at baseline, at 3 M, at 6 M, at 12 M and once a year there after). A p value less than 0.05 was considered significant.

## Results

Out of the 70 originally recruited AS patients, half (35 = 50 %) continued to take infliximab for the entire 5-year study period. At baseline, higher BASDAI and BASFI scores and mean CRP levels confirming active disease was found in both those who later had a positive response to the therapy (responders) as well as in those who did not (non-responders). Twenty-nine (82.9 %) of the responders were male (mean age 50.4 ± 12.5; mean disease duration 17.9 ± 10.6 years); the majority of the non-responders were female (34.3 % vs 17.1 %). At baseline (0 M) the characteristics of the responders were similar to those of the non-responders -except for prednisone intake and the VASg score -, which were higher in the latter with respect to the former (Table 1)*.*

**Table 1:** Demographic, clinical, and biohumoral characteristics at baseline of the 70 AS patients who were treated with infliximab treatment and classified into two groups: those still taking the drug (responders) or not taking the drug (non-responders) at the end of the five year period

	Responders	Non-responders	p^*^
N	35	35	
Male, N (%)	M 29 (82.9 %)	M 23 (65.7 %)	ns
Age (years)	50.3 ± 12.5	48.5 ± 11.0	ns
DiseaseDuration (years)	17.9 ± 10.6	16.3 ± 9.3	ns
MTX intake, N (%)	15 (42.9 %)	12 (34.3 %)	ns
Sulfasalazine, N (%)	3 (8.6 %)	5 (14.3 %)	ns
Prednisone intake, N (%)	5 (14.3 %)	14 (40 %)	<0.005*
Uveitis, N (%)	1 (4.1 %)	1 (4.1 %)	ns
IBD, N (%)	1 (4.1 %)	0	ns
NSAIDS, N (%)	29 (82.9 %)	27 (77.1 %)	ns
ESR (mm/H)	21.5 ± 18.3	31.0 ± 25.1	ns
CRP (mg/dL)	1.9 ± 1.6	1.7 ± 1.5	ns
BASDAI	59.5 ± 15.3	59.9 ± 19.6	ns
BASFI	47.1 ± 16.8	52.2 ± 25.1	ns
BASMI	5.9 ± 1.3	5.4 ± 1.6	ns
VAS	64.9 ± 16.9	62.9 ± 18.8	ns
VASg	50.5 ± 21.4	61.1 ± 19.0	0.05*

*N* number, *MTX* Methotrexate, *IBD* Inflammatory Bowel Diseases, *NSAIDs* Non Steroidal Anti-Inflammatory Drugs, *ESR* Erytrocyte Sedimentation Rate, *CRP* C-Reactive Protein, *BASDAI* Bath Ankylosing Spondylitis Disease Activity Index, *BASFI* Bath Ankylosing Spondylitis Functional Index, *BASMI* Bath Ankylosing Spondilitis Metrology Index, *VAS* Visual Analogue Scale pain, *VASg* Visual Analogue Scale global disease activity

Data expressed as mean ± standard deviation, unless otherwise indicated

*Mann Whitney test

**Fig. 1:**
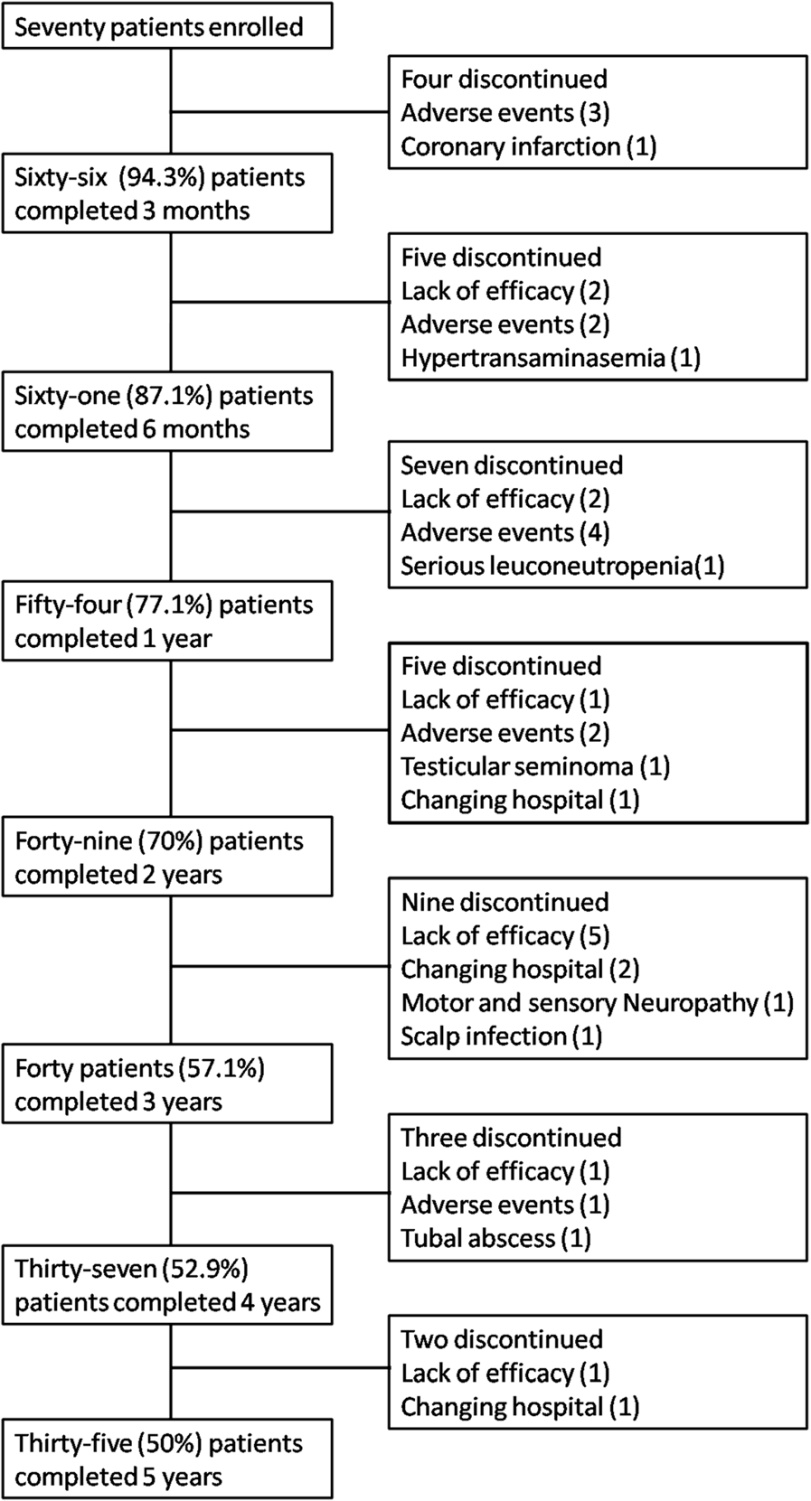
Flow chart of the five year period

**Fig. 2:**
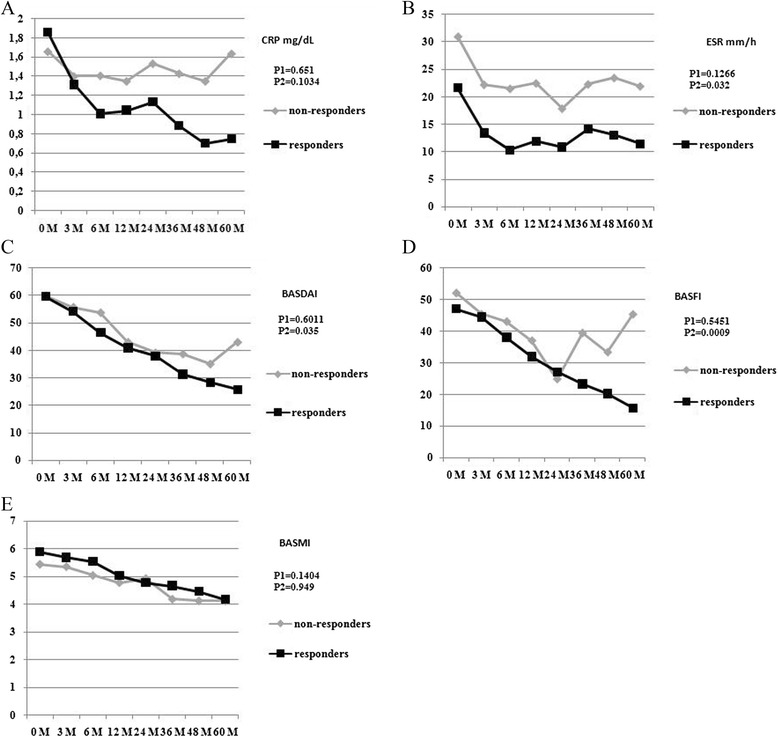
Serological, clinical and functional parameters during the 5-year study period. **a** CRP, **b** ESR, **c** BASDAI, **d** BASFI, **e** BASMI in completers (*n* = 35) and in discontinuers (*n* = 35). Mann Whitney test: p1 = M0 responders vs M0 non responders; p2 = M60 responders vs M60 non-reponders

**Fig. 3:**
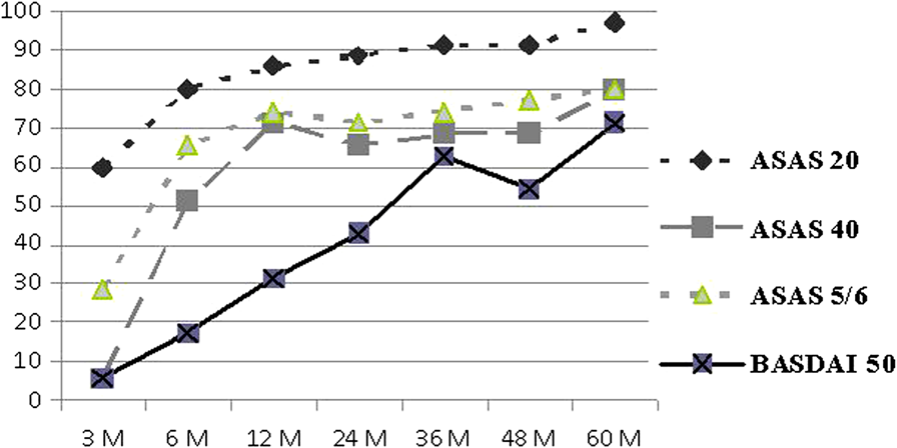
Clinical outcome in responders (*n* = 35;50 %) over the five year period (expressed in percentage %). M = months; ASAS 20 = Ankylosing Spondylitis Assessment Study Group 20 %; ASAS 40 = Ankylosing Spondylitis Assessment Study Group 40 %; ASAS 5/6 = Ankylosing Spondylitis Assessment Study Group 5/6; BASDAI 50 = Bath Ankylosing Spondylitis Disease Activity Index 50

Half (35/70) of the patients discontinued therapy due to insufficient response or adverse events to infliximab during the 5-year study period. There were instead 54 (77.1 %) patients still taking infliximab at one year, 49 (70 %) at 2 years, 40 (57.1 %), at 3 years and 37 (52.9 %) at 4 years. The causes leading patients to withdraw were evenly distributed over the 5-year study period (Fig. 1) and included: therapeutic ineffectiveness (in 12 = 34.3 %), adverse events - such as injection site reactions, headache, hypertensive crisis, tachycardia, vertigo, abdominal pain - (in 12 = 34.3 %), several site effects and in 4 (11.4 %) cases the patient moved away (Fig. 1). There was a statistically significant improvement in all of the indices over the study period in the responders. The patterns of the various study parameters over the 5-year period are outlined and compared in Fig. 2. There was a significant decrease in the BASDAI, BASFI, ESR indices in the responders with respect to the non-responders: at the end of the study the BASDAI was, in fact, 25.8 ± 18.5 and 43.1 ± 21.7 (*p* = 0.0347), the BASFI was 15.8 ± 12.2 and 45.5 ± 26.1 (*p* = 0.0009), the ESR was 11.5 ± 8.74 and 21.9 ± 15.8 (*p* = 0.0323), respectively. There was also a slight but not statistically significant improvement in the CRP and BASMI values in the responders (Fig. 2). At 3 M, the BASDAI and the ASAS scores in the responders were already beginning to show an initial positive response and at 6 M there was a significant, clear improvement which was maintained throughout the 5-year period (Fig. 3). After 5 years of treatment 25 (71.4 %) achieved 50 % improvement in the BASDAI, 34 (97.1 %) attained the ASAS 20 %, 28 (80 %) attained the ASAS 40 %, and 28 (80 %) achieved the ASAS 5/6. Clinical improvement was also associated to lower ESR and CRP: CRP levels fell from 1.9 ± 1.6 at 0 M to 0.8 ± 1.5 at 60 M, and ESR levels fell from 21.7 ± 18.2 at 0 M to 11.5 ± 8.7 at 60 M (Fig. 2 and Table 2).

**Table 2:** Serological, clinical, and functional parameters during the five year study period

	Variable	ESR	CRP	BASFI	BASDAI	BASMI	VAS	VASg
	(mean ± SD)							
Responders	0 M	21.7 ± 18.2	1.9 ± 1.6	47.1 ± 16.8	59.5 ± 15.3	5.9 ± 1.3	64.9 ± 16.9	50.5 ± 21.4
	3 M	13.5 ± 12.8	1.3 ± 1.9	44.5 ± 17.5	54.2 ± 17.3	5.7 ± 1.4	61.3 ± 15.1	45.4 ± 22.1
	6 M	10.4 ± 8.8	1.0 ± 1.4	38.1 ± 22.2	46.5 ± 20.1***	5.6 ± 1.3	55.3 ± 12.4*	40.6 ± 21.6*
	12 M	11.9 ± 12.4	1.1 ± 1.4	32.1 ± 20.1**	40.9 ± 19.4*	5.1 ± 1.6***	41.1 ± 11.9*	35.6 ± 19.9*
	24 M	10.9 ± 10.2	1.2 ± 1.9	27.2 ± 17.4*	38.9 ± 20.9*	4.8 ± 1.4*	37.8 ± 10.9*	32.4 ± 17.8*
	36 M	14.2 ± 12.6	0.9 ± 1.1	23.4 ± 15.3*	31.4 ± 18.1*	4.7 ± 1.4*	36.2 ± 10.1*	30.5 ± 18.7*
	48 M	13.1 ± 8.1	0.7 ± 1.6*	20.4 ± 13.9*	28.2 ± 18.8*	4.5 ± 1.3*	32.6 ± 12.1*	25.2 ± 19.6*
	60 M	11.5 ± 8.8	0.8 ± 1.5*	15.8 ± 12.2*	25.8 ± 18.6*	4.2 ± 1.4*	30.8 ± 13.2	20.3 ± 19.8
	*p*	0.087	<0.0001	<0.0001	<0.0001	<0.0001	<0.0001	<0.0001
Non-responders	0 M	31.0 ± 25.1	1.6 ± 1.5	52.2 ± 25.1	59.9 ± 19.6	5.4 ± 1.6	62.9 ± 18.8	61.1 ± 19.0
	3 M	22.2 ± 22.6	1.4 ± 1.7	45.5 ± 21.9	55.7 ± 19.1	5.3 ± 1.6	57.2 ± 22.3	59.2 ± 22.9
	6 M	21.5 ± 7.1	1.4 ± 1.6	43.2 ± 23.1	53.7 ± 19.9	5.0 ± 1.4	55.4 ± 20.6	60.5 ± 21.1
	12 M	22.5 ± 20.4	1.4 ± 1.4	37.1 ± 24.9	23.0 ± 43.9	4.8 ± 1.6	47.8 ± 21.3	49.2 ± 20.0
	24 M	17.9 ± 12.9	1.5 ± 3.1	24.9 ± 15.6	39.1 ± 25.6	4.9 ± 1.4	49.7 ± 22.2	55.5 ± 22.0
	36 M	22.3 ± 14.1	1.4 ± 1.3	39.6 ± 27.9	38.8 ± 26.9	4.2 ± 2.2	50.2 ± 18.8	52.4 ± 19.7
	48 M	23.5 ± 22.7	1.4 ± 2.1	33.4 ± 25.5	35.1 ± 23.3	4.1 ± 1.4	57.3 ± 17.4	60.6 ± 20.1
	60 M	21.9 ± 15.8	1.6 ± 2.3	45.5 ± 25.1	43.1 ± 21.7	4.1 ± 1.5	58.2 ± 18.5	65.1 ± 19.8

*ESR* Erytrocyte Sedimentation Rate, *CRP* C-Reactive Protein, *BASDAI* Bath Ankylosing Spondylitis Disease Activity Index, *BASFI* Bath Ankylosing Spondylitis Functional Index, *BASMI* Bath Ankylosing Spondilitis Metrology Index, *VAS pain* Visual Analogue Scale pain, *VASg* Visual Analogue Scale global disease activity

Data are expressed as mean ± SD. The Friedman repeated measures test and Dunn's multiple comparison test were used * *p* < 0.0001 vs T0, **p < 0.001 vs T0, ***p < 0.01 vs T0

Interestingly, only two patients (2.8 %) had uveitis, one of these responded to inflximab treatment and the other did not. The frequency of uveitis episodes fell from 3 times/year to 1 time/year for the first year of treatment and to 0 time/year for the following years in the responder. Only one non-responder had a single episode of uveitis in the entire study period and he was successfully treated with topical steroids. Only one of the patients had AS associated to inflammatory bowel disease (Crohn disease) and was treated with infliximab; his response both with regard to articular as well as bowel symptoms was good (Table 1).

Forty-five (64.3 %) of the patients developed infections, the severity of which ranged from mild (requiring temporary interruption of treatment) to severe (requiring permanent suspension of treatment) (Table 3).

**Table 3:** Data concerning infections in the patients studied during the five year study period

Most frequently reported infections in 5 years study population (*n* = 70 patients)	N (%)
Upper respiratory tract infections (rhinosinusitis, pharyngitis, laryngitis, colds)	13 (18.6 %)
Bronchitis/bronchopneumonia	6 (8.6 %)
Conjunctivitis eye disorders	5 (7.1 %)
Influenza episodes	4 (5.7 %)
Diarrhea	4 (5.7 %)
Urinary infections	4 (5.7 %)
Otitis	2 (2.8 %)
Severe skin infections	2 (2.8 %)
Dental infections	2 (2.8 %)
Vaginitis	1 (1.4 %)
Herpes labialis	1 (1.4 %)
Tubal ascess	1 (1.4 %)

**Fig. 4:**
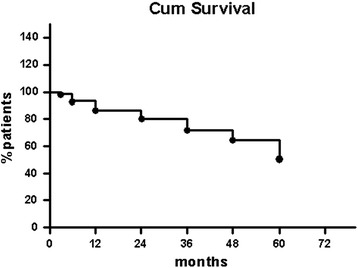
Infliximab survival (percentage responders) over the 5-year study. “Cum” survival = cumulative survival

Drug survival was calculated as the number of days that the individual patients took the drug. The start date was the day the first dose was taken and stop date was the day treatment was interrupted. Temporary interruptions *e.g.,* due to infections or surgery of ≤3 months’ duration were acceptable according to our study protocol. In two cases the severity of infection (severe scalp infection and tubal abscess) necessitated permanent discontinuation of infliximab treatment (Fig. 1). Infliximab survival (percentage of responders) over the 5 study period is outlined in Fig. 4. Thirty-one (44.3 %) of the 35 responders were able to prolong the time interval between infusions (the mean time interval was 7.5 ± 4.1 weeks).

## Discussion

In agreement with other reports, these data confirm that infliximab therapy is safe, tolerable and effective in AS patients [10, 11, 16–22, 27, 28]. The rapid stable, substantially positive therapeutic effect of infliximab treatment in most patients with active AS has recently been demonstrated by an open-labeled multicenter study which described a statistical improvement in the ASAS criteria in 84.8 % of the patients who completed the trial [29]. Data confirming infliximab’s efficacy and safety have also been reported by some registry-based studies. The Czech national ATTRA registry in fact, found a longer adherence to anti-TNFα therapy due to clinical improvement and a prolonged, satisfactory response, in particular to infliximab, in AS with respect to RA patients [30]. The TRASD-IP registry also described the positive effect that infliximab treatment had on patients’ quality of life [31]. According to recent results based on the Danish DANBIO registry, out of 1436 AS patients who started anti-TNFα treatment, 30 % later switched to a second and 10 % to a third biological drug [32]. The authors also reported that after 2 years of treatment 52 % of the switchers and 63 % of the non-switchers achieved treatment response and that the majority of switchers were women [33].

Our study also confirmed that the female gender seems to be a negative predictor of drug response and disease outcome. Other values which differed at baseline between the responders and the non-responders were the global disease activity score (VASg) and steroid intake, which can be interpreted as additional negative predictors.

In accordance with other reports, the main outcome measures of our study were disease-related clinical parameters and subjective indices, such as the BASDAI, the BASFI and the BASMI scores. An improvement was noted in disease activity, mobility, and function that was largely maintained in our responders over the 5-year study period. While improvement peaked at 6 M, it was already noticeable at 3 M. In contrast to Baraliakos et al. who described a slight functional decline during long observations periods [27], a modest although continuous improvement was noted in our patients throughout the study period (Fig. 2). As far as Baraliakos’ patients are concerned [27], the decline that was noted may have been linked to a decrease in the proportion of patients in partial remission noted during the last year of that study and the progressive structural damage that was taking place over the study period [27, 33]. Interestingly, the patients with a slight increase in BASFI scores at the end of the study were found to have more radiographic damage [27]. The significance of radiographic damage in AS has recently been studied by some authors who reported that anti-TNFα does not seem to have important effects on radiographic progression in these patients [34]. It is impossible to exclude the existence of a bias linked to other concurrent treatments, such as NSAIDs, physical therapy and/or rehabilitation. Intensive rehabilitation seems, in fact, to have a synergistic effect in AS when it is associated to TNFα, producing positive effects on pain, function, quality of life and disability [33, 35]. In our study, the fall in the responders in CRP and ESR levels, parameters considered important inflammatory markers, followed a decrease in disease activity indices. BASFI and BASDAI scores showed ongoing improvement throughout the 5-year study period (Table 2, Fig. 2). The persistent effectiveness of infliximab, already noticeable at 6 M, was confirmed by the BASDAI 50, the ASAS 20, the ASAS 40, and the ASAS 5/6 scores also at the end of the study. Our results are in agreement with those described by Danish investigators who found a rapid, sustained decrease in disease activity in AS patients being treated with TNFα inhibitors within 6 months of treatment onset [36]. The infliximab survival rate was high, in our study reaching 50 %. Interestingly, those same parameters showed an opposite trend in the non-responders: after an initial improvement, the BASFI and BASDAI began to rise in the non-responders while the ESR and PCR values remained at higher although stable levels compared to those in the responders (Fig. 2). Our data support the hypothesis that disease activity indices and inflammatory markers are predictors of patients’ clinical response to anti-TNFα treatment and could be useful when patients with a negative response are being evaluated for discontinuation.

Therapy in our patients was mostly discontinued for reasons linked to infusion-related reactions (*n* = 12; 34.3 %), loss of efficacy (*n* = 12; 34.3 %) or to several side effects (*n* = 7; 20 %) rather than to minor side effects/mild infections. In accordance with data collected by our center regarding SpA patients taking other biologic agents, some of the participants in the present study developed mild infections [37]. Serious infections were, instead, noted in 5 of the As patients participating in the present study: two developed bronchopneumonia requiring hospitalization, two developed serious skin infections (of the scalp and/or severe folliculitis), and one developed a tubular abscess with serious vaginitis. After discontinuing infliximab therapy for a brief period, all except for the two patients who presented infection of the scalp and tubular abscess were able to resume treatment. The drug’s safety was similar to that described in previous studies [27, 38, 39]. Our results have confirmed that when first line treatment fails switching to another biotechnological drug can be an effective solution and they are in agreement with our previous retrospective study conducted on a total of 1619 SpA patients treated with infliximab (35.3 %), etanercept (43.7 %) and adalimumab (20.9 %) [40]. Out of the switchers, there were only 19 AS patients receiving infliximab as their first anti-TNFα drug and that study showed that patients who failed to respond to the first agent often responded to a second-line drug regardless of the reason for switching.

## Conclusion

Study results indicated that infliximab therapy elicited a beneficial, long-term, safe effect in AS patients over the 5-year study period. Patient amelioration was confirmed by a marked, persistent improvement in both subjective and objective disease activity. Study data also suggested that the female gender, elevated prednisone intake (>5 mg/day), high VASg levels at baseline, no fall in inflammatory indices, persistently high BASFI and BASDAI levels can all be considered predictors of future anti-TNFα treatment discontinuation. This study has, of course, some limitations: most importantly, the study population is small. In addition, since the patients needed to fulfill the New York criteria, only those individuals with a relatively advanced disease form were included. As a consequence, its results cannot be applied to patients with early, non-radiographic axial SpA. The study did produce one original finding: that simple clinical/laboratory markers of disease activity can already predict at baseline which AS patients are most likely to have a poor response to treatment. This information could, of course, be useful to specialists who are managing AS patients and further studies are warranted to confirm these data.

### Key messages

- The clinical impact of AS is severe and its progression leads to disability
- With appropriate treatment, patients with AS can live relatively normal, independent lives
